# 

**Published:** 1848-06

**Authors:** 


### INDEX TO VOLUME FOURTH.

*Note.*—Original articles may be found by referring, in the index, to the initials of the names of authors. For other articles, generally, look for the first letters of the names of the subjects of which they treat.

Page.

Association, American Medical, meeting of, at Baltimore, May 2d, 1848 17

" " " transactions for 1848, 421

" " and Medical Colleges, 770

Association of Southern Central New York, 609

" Michigan State Medical, 773

Aneurism Popliteal successfully treated by compression, by J. Knight M. D. and D. A. Tyler M. D. 38

Abscess, case of, terminating in profuse hemorrhage, 43

Anatomical preparations, new fluid for preserving the color of, 114

Association of Superintendents of Insane Institutions, meeting of, 172

Arsenic, poisoning by, magnesia antidote, 238

" " treated by magnesia, 309

" " trial for poisoning by, 630

Anæsthetic agents, use of, 311

Asylum, Lying-in, N. Y. annual report, 321

" Lunatic, Utica, annual report, 662

Anthropo-toxicologia, cases with remarks by Dr. Lavender, 438

Aneurism of aorta and rupture within pericardium, 580

Bartlett on Fevers, Review, 26

Blake, James M. D. on action of medicines, 48

Buffalo, University of, medical department, Commencement 62

" " College edifice, 201

" " change in lecture term, 702

" " Commencement, 764

" " History of, 765

Buffalo Med. Association, Regulations of, on preliminary med. education, 62

Bell and Stokes, lectures on the theory and practice of Physic, notice of, 117

Bird's elements of Natural Philosophy, notice of, 118

Burrows on cerebral circulation, notice of, 119

Blakiston on diseases of the chest, 122, 277

Bowditch, young stethoscopist, 123

Belladonna, extract, poisoning by, case 125, 200

" in incontinence of urine, 761

Blood, pathology of, in inebriates, 181

Bed-sores, simple mode of preventing, 186

Buffalo Hospital of Sisters of Charity, 325, 458, 659, 775

" marriages and deaths in, 389

Burwell, G. N., M. D, fifteen cases of midwifery in which chloroform was administered, 331

Page.

Black Eagle, 390

Bryan, Professor James, translation of report of the Concours relative to apparent death, 463

" " " essay on strictures of urethra, 707

" " " trans. of report on tamponing, 730

Beck, Prof. J, B. on infant therapeutics, notice of, 516

Blackwell, Miss Elizabeth, M. D. thesis on ship fever, 523

Blackwell, Miss Elizabeth, M. D. 584

Bedford, Prof. lecture, 708

Cholera in Europe and Asia, 55

" Epidemic, 329, 502

" " progress of, 390

" in granite regions, 499

" vapor baths in, 500

" chloroform in, 500

" editorial remarks on, 504

" statistics of, 52

" report by Dr. Whiting, 561

" at New Orleans, 578, 517

" at New York, 520, 703

Constipation, remarkable case of, 113

Cartwright, Dr. statistics of Quackery, 104

Collodion controversy, 129

" mode of preparing, 197

" in diseases of the skin, 573

Coventry, Prof. C. B. report on epidemic cholera, 155, 201

Coventry, Prof. C. B. on cholera, report of, 712

Coventry, Prof, C. B. on cholera, 737

Churchill's midwifery, notice of, 191

Connecticut Medical Society, 196

Chloroform, Prof. Murphy on, 227

" external use of, by Nunnelly, 236

" death from, 626

" discoverer of, 696

Camphor, new vehicle for solution, 235

Colculus urinary, extraction by a novel method, 303

Cleveland Med. College anouncement, 325

Curiosities of medical literature, 387

Colegrove, Bela H. M. D., amputation ot the wrist. chloroform, 388

Curiosities of medical experience, case, 393

Clerical and Medical Professions, relations between. 431

Chiniodine, 434

Channing on Etherization in childbirth, notice of, 515

Combe's guide to health, notice of, 519

Convention of delegates from Western schools, 521, 650

Changes, editorial, 579

Croup treated by cauterization, 636

Capillary circulation, by Dr. Dowler, 664

Page.

Conjunctivitis, Prof. Parker on, 695

Cod-liver oil, 696, 700

Cyanosis by Rokitansky, 749

Camphor and chloroform mixture, 762

Deaths, marriages and births in State of N. Y., 41

Drugs, inspection of, 61

Death, singular case of, 789

Dysentery, prescription for, 321

Dysentery treated by injections of nitrate of silver, 628

Dysmenorrhoea, prescription for, 380

Druit's surgery, by Sargeant, notice of, 516

Dunglison's medical lexicon, notice of, 518

Dowler, Bennett, M. D., 581

Drug law new, effects of, 629

Expectoration, diagnostic value of, by Andral, 108

Ely Dr. on nursing sore mouth, 232

Erysipelas, treatment of, 375

Editorial changes, 385, 776

Erie Co. Superintendents of Poor, report of, 446

Eve, Prof. case of calculus in Perineum and bladder, 607

Fever treated by cold water, by Dr. Gill, 44

Fever, yellow, at Staten Island N. Y. 322

Fever, cases of, with remarks by editor, 355

Fever, case of common, continued (typhoid) by editor, 487

Fenner on dislocation of femur and fracture of cranium, 184

Flies, lavae of, ejected from stomach, 498

Flint, Dr. A., clinical lecture, on the diagnosis of Pulmo-tuberculosis, 587

Flint, Dr. A., medical cases at Buffalo Hospital of Sisters of Charity, 743

Green, Dr. C. on bending and partial fracture of radius 168

Green, Dr. C. on preparing collodion, 197

Green, Dr. C. Med. notes and observations, 721

Gestation, prolonged case of, 182

Griffith on blood and urine, notice of, 191

Griffith's dispensatory, 192

Gutta Percha, medical employment of, 239

Gun Cotton, ethereal solution of, 244

Geneva Medical College 393

Green on Croup, notice of, 482

Gardener's medical chemistry, notice of, 517

Glossitis, case of, 611, 615

Graham, Dr., 649

Grant, Prof., Introductory lecture, 707

Gooch on diseases of women, notice of, 708

Graves clinical medicine, notice of, 709

Hamilton, Dr. F. H., Surgical dispensary reports, 23, 83

Hamilton, Dr. F. H., Resignation of, at Geneva, 201

Hamilton, Dr. F. H. Rhinoplasty, Blepharoplasty and Cheiloplasty in same patient, 459

Hamilton, Dr. F. H., three cases of Harelip, 306

Page.

Hamilton, Dr. F. H. Fracture tables, 615

" " Lithotomy in child, 735

" " " in adult, 736

" " Report of prosecution for mal-practice, 274

" " Plastic operations, 344

Hall, Marshall M. D., on Spasmo-paralysis in infants and adults, 169

Homeopathists, veracity of, 194

Headack on, by Dr. Murphy, 228

Hernia, Petit's operation for, 230

Haematuria, case of, by editor, 272

Hemorrhage uterine, 380

Hair cutting, hygienic effects of, 445

Hospitals, History of. 491

Hospital, Bellevue, 692

Head, passage of iron rod through, 493, 584

Hydrophobia treated with chloroform, 497

" case of, 638

Harrison's Anatomy, notice of, 517

Hard, H. M., M. D., on injection of iodine into suppurating cavities, 531

Heart, needle found in, after death, 637

Hysterotomy vaginal, case, 640

Heart-clot, by Prof, Meigs, 865

Heart, fibrinous concretion in, 701

Hydropathy, Hood's illustration of, 701

Handbills for cure of diseases 713

Hiccups, cure for, 763

Isopathy, 130

Iron per-sesqui-nitrate, medicinal effects of, 187

Ingalls on ship-fever, 193

Idiots, education of, 215

Intoxication by G. Corfee, 234

Insane asylums, 248

Iron, citrate of, syrup, 381

Insurance, life, and irregular practitioners, 711

Jerks 242

Jones on diseases of Onondaga Co. 758

Kreasote in Erysipelas, 189

Kneeland, Jonathan, M. D., case of hemorrhagic effusion into the peritoneal cavity, 419

Kirtland's, Prof. Jared P., introductory lecture, notice of, 511

Knapp, Prof., address 708

Legal responsibility of discontinuing medical attendance 60

Lecture terms, extension of, 127, 329

" " length of, in different colleges 328

Lockwood, Dr. T. T., on Hornet's nest, 128

Lectures, daily reduction of, in medical colleges 324

Lallemand on spermatorrhea, notice of, 382

Lee, Prof. C. A. clinical lecture on Aphonia and stammering, 411, 456

Lee, Prof. C. A. clinical lecture on diseases of reflected and sympathetic nervous influence, 533

Lunatic, Hospitals reports of, 704

Lectures, Popular Hygienic and medical, 777

Page.

Medical Dispensary Univ. of Buffalo, Report of cases by the Editor, 86

Muller's Physics and Meteorology, notice of, 118

Malpractice, report of trial for, Wm. Tims vs. James P. White 131, 193

Malpractice reported by Dr. Hamilton 274

March, Prof., letter from Paris, 176

Meigs, J. Forsyth M. D., on diseases of children, notice of, 192

M'Clellan's Surgery, review of, 209, 347

Means on poison of venemous reptiles, 219

Medical men, political distinction of, 246

Meningitis, cerebro spinal Epidemic, 299

Meigs, Prof. C. D. case of Pseudo-membronous Laryngitis, 305

Meigs, Prof. C. D. Letters on Females notice of, 318

Meachem, T. G. M. D. on dysentery in East Bloomfield, N. Y. 395, 517, 583

Medical schools, new, 454

" " in Russia 643

Medical education in Turkey 641

Medical periodical literature, American, encouragement of, 643

Medical College, National, 646

Morton, W. T. G. report of Dr. Edwards on memorial to House of Representatives, 706

Manley, James R., discourse before the N. Y. academy of medicine. 708

Morton's system of anatomy, 708

New York University, Medical department, annual announcement of lectures, session, 1848—9, 56

New York, state medical society, Vol. VII. Part II, 64

Narcotic necessaries of life, 245

Nostrum trade, repudiation of, by an apothecary, 393

Nitrate of lead as a disinfectant, 499

Neil and Smith's compendium, notice of, 515

Obituary, Dr. A. G. Grow, 714

" George Sprague, 392

Pessary, new article for, 55

Physicians and clergy, 259

Pennsylvania college, annual announcement, 263

Pennsylvania University of, Med. Dep. report on, 265

Pharynx angina folliculosa, by M. Chomel 306

Parker, Prof., on Tobacco, Tea and Coffee, 308

Practical men, so called, 379

Papin, T. S., letter from Paris 501

Paul, Z.. M. D., on treatment of fractures, 606

Placenta, manual delivery of, by Dr. Smith, and Editor, 682

Phthisis and marsh-fever, 754

Professorship vacant, 775

Quackery, victim to, 443

Page.

Quinine, spurious, 761

Rattlesnake, death by bite of, 175

" poisoning by, treatment of, 203

" iodine, in poisoning by, 575

Rheumatism, nitrate of potash in, by Prof. Gilman, 442

Revaccinations in the Prussian army, 498

Reid, Dr. W. W. reminiscences of cholera in 1832, 550

Scott, W. K., M. D., case of vaginal hysterotomy, 37

Stevens, Alexander H. M. D. address on the food of plants, 61

" " " address before state medical Society, 707

Strychnia, recovery from a poisonous dose of, 111

Smart-weed in salivation and stomatitis, 183

Surgery at a discount, 190

Smith, Wm. G. on the medical treatment of cataract, 267

Sargeant on bandaging and minor surgery, notice of, 320

St. Louis University, med. dept., 372

Stokes, Wm. H. M. D., rejoinder to Dr. Brigham, 372

" " " vs. American Journal Insanity, 567

Stuart on the lungs, 385

Shipman, Prof. A. B., valedictory, notice of, 519

Scabies, treatment of, 630

Smith, Prof. discourse, 707

Society, State Medical, 710

Stomach separation from œsophagus, 762

Treat, Dr. Wm., old physic and young physic, 1

Taylor on poisons, notice of, 117

Trowbridge, Dr. Josiah, Portrait of, 127

" " on Rattlesnake bite, 203

Tracheotomy successfully performed in case of inflamation of fauces & glottis, 571

Tetanus idiopathic in horse treated with chloroform, 627

Taylor, Gen. compliment by state med. society, 714

Vaccine virus depot, 384

Venereal disease, new treatise on, 384

Varicocele, new operation for, by Prof. Gross, 576

Warren, J. Mason M. D., operation for fissure of soft and hard palate, 24 cases, 64

Wright on human existence, notice of, 123

White, Prof. James P. case of puerperal convulsions, treated with chloroform, 206

" " on obstetrical forceps, 715

Whitehead on abortion, notice of, 314

Wood, Prof. and British Provincial med. and surg. association, 503

Yandell, Prof. L. P., Introductory lecture, notice of, 518

" " on Etherization, 707

☞ Our readers will perceive a change in the typographical appearance of the Journal. It is printed upon new long primer type, which allows of more matter per page, as well as presents a better appearance. Some delay in issuing the June number has been rendered neccessary by this change. We hope soon to make our issues promptly on the first day of each month. For further information respecting the present volume, see circular on the cover.

